# Supplementation with Whey Protein, Omega-3 Fatty Acids and Polyphenols Combined with Electrical Muscle Stimulation Increases Muscle Strength in Elderly Adults with Limited Mobility: A Randomized Controlled Trial

**DOI:** 10.3390/nu12061866

**Published:** 2020-06-23

**Authors:** Claire Boutry-Regard, Gerard Vinyes-Parés, Denis Breuillé, Toshio Moritani

**Affiliations:** 1 Nestlé Research, 1015 Lausanne, Switzerland; denis.breuille@rdls.nestle.com; 2 Nestlé Health Science S.A., 1066 Epalinges, Switzerland; Gerard.VinyesPares@nestle.com; 3 Laboratory of Applied Physiology, Graduate School of Human and Environmental Studies, Kyoto University, Kyoto 606-8501, Japan; moritani.toshio.76s@st.kyoto-u.ac.jp

**Keywords:** aging, muscle size, muscle strength, gait speed, whey protein, omega-3 fatty acids, curcumin, rutin, electrical muscle stimulation

## Abstract

Age-related sarcopenia is a progressive and generalized skeletal muscle disorder associated with adverse outcomes. Herein, we evaluate the effects of a combination of electrical muscle stimulation (EMS) and a whey-based nutritional supplement (with or without polyphenols and fish oil-derived omega-3 fatty acids) on muscle function and size. Free-living elderly participants with mobility limitations were included in this study. They received 2 sessions of EMS per week and were randomly assigned to ingest an isocaloric beverage and capsules for 12 weeks: (1) carbohydrate + placebo capsules (CHO, *n* = 12), (2) whey protein isolate + placebo capsules (WPI, *n* = 15) and (3) whey protein isolate + bioactives (BIO) capsules containing omega-3 fatty acids, rutin, and curcumin (WPI + BIO, *n* = 10). The change in knee extension strength was significantly improved by 13% in the WPI + BIO group versus CHO on top of EMS, while WPI alone did not provide a significant benefit over CHO. On top of this, there was the largest improvement in gait speed (8%). The combination of EMS and this specific nutritional intervention could be considered as a new approach for the prevention of sarcopenia but more work is needed before this approach should be recommended. This trial was registered at the Japanese University Hospital Medical Information Network (UMIN) clinical trial registry (UMIN000008382).

## 1. Introduction

The world population is aging rapidly [1]. Sarcopenia and frailty are detrimental health-related events highly prevalent in elderly adults [2,3]. Both are associated with a loss of functional capacity and a dramatic decrease of quality of life. Multiple, inter-related factors contribute to the progressive loss of muscle mass and function: lifestyle changes [4], metabolic changes, such as anabolic resistance [5] insulin resistance, inflammation [6,7], oxidative stress or mitochondrial dysfunction [8].

One well-known nutritional solution for sarcopenia is protein supplementation. An association between protein intake and muscle mass [9] or muscle functionality was established [10,11,12]. Current experts’ suggestion for healthy older adults is to consume an average daily protein intake of 1.0 to 1.2 g/kg body weight (BW)/d [13] for preserving muscle functionality. On top of this, there are other nutritional approaches that have been proposed to manage oxidative stress, to decrease low-grade inflammation and/or to improve insulin sensitivity and increase the anabolic effect of nutrients in order to not focus only on muscle protein synthesis. Supplementation with polyphenols (curcumin and rutin) and antioxidants (vitamin E, vitamin A, zinc and selenium) partially restored the anabolic effect of leucine on muscle protein synthesis rate in rats [14,15]. Only one clinical study looking at the beneficial effect of polyphenols on muscle physiology is available. Curcumin supplementation combined with exercise was shown to improve physical performance, such as walking speed of healthy elderly adults [16]. There are a few studies which demonstrated that omega-3 polyunsaturated fatty acids (ω3) supplementation was able to increase the sensitivity of muscle protein metabolism to anabolic factors [17] in older adults and to improve muscle mass and functionality in elderly adults [17,18,19,20].

The beneficial impact of resistance or endurance training on muscle mass and function is also highlighted in many studies [21,22]. However, physical exercise may be difficult to carry out in frail or bedridden elderly adults. One alternative is to use electrical muscle stimulation (EMS) applied as “involuntary” exercise [23,24]. EMS was reported to provide identical or even greater benefits compared to voluntary muscle training during a phase of reduced activity [25] or after an immobilization period [26] and to show positive effects on muscle mass [27], muscle strength [28,29], balance [30] and functional performance [31,32] in elderly adults.

Thus, a multimodal approach combining nutrition and exercise induced by EMS may provide an innovative treatment for limiting the development of sarcopenia with aging. Until now, no studies have been carried out investigating the combined approach of nutrition and EMS. Currently, we hypothesize that the effect of EMS could be improved with the consumption of a whey-based nutritional intervention with a specific blend of ingredients containing polyphenols and ω3 fatty acids (exhibiting antioxidant and anti-inflammatory activities) to further enhance the benefits of the whey-based nutritional intervention.

## 2. Materials and Methods

### 2.1. Participants

The clinical study was performed at the Momoyama social welfare center for the elderly in Kyoto (Japan). The study protocol received the approval of the ethical committee from Kyoto University (ethics approval number 23-H-15) and was conducted in accordance with the Declaration of Helsinki and in compliance with the Japanese ethical guidelines for clinical studies. Forty-one participants (33 females, 8 males) with mobility limitations were recruited based on the following inclusion criteria: mobility limitations according to the long-term care insurance (LTCI) system of Japan, age between 60 and 90 years and gait speed below 1.5 m·s^−1^. All participants included in the study were “free-living” people without any support or classified in Care support level 1, Care support level 2, Long-term care level 1 (LTC1) and Long-term care level 2 (LTC2) according to the LTCI system. This is a national system in Japan including everyone from age 65 and above used to determine the amount of services covered by the insurance (institutional and community-based services) [33]. The LTCI system uses the Kihon Checklist, a self-administered questionnaire consisting of 25 items in seven categories: physical strength, nutritional status, oral function, socialization, memory, mood and lifestyle [34,35]. Participants were included after a thorough medical examination (orthopedic and medical questions) made by a physician and a care manager to determine their suitability to participate in an EMS program.

Exclusion criteria were as follows: body mass index (BMI) > 25 kg/m^2^, regular exercise activity, history of neuromuscular impairments, currently participating or having participated in another clinical trial in the 4 weeks prior to the beginning of the study, consumption or planning to consume dietary supplements such as free amino acids, antioxidants or supplements specifically designed for malnourished elderly people, treatment adherence and serious chronic disease (e.g., chronic kidney disease, liver cirrhosis, heart failure, cancer, major systemic diseases or any health impairment as deemed clinically significant by the investigator).

All participants received detailed information on the protocol and gave their (or a legal representatives) written informed consent to participate in the study.

### 2.2. Study Design

The study was a single center, parallel, double-blind, randomized-controlled trial registered in the Japanese UMIN (University hospital Medical Information Network) clinical trial registry (UMIN000008382 at www.umin.ac.jp). The aim of this study was to assess the effects of a combination of EMS and a whey-based nutritional supplement (with or without polyphenols and fish oil-derived ω3 fatty acids) on muscle function and size in a physically compromised elderly population. The primary outcome measure of this study was the estimated change of the thickness of the thigh (rectus femoris) and calf (gastrocnemius) muscle using ultrasonography. In addition, gait speed, knee extension strength and nutritional assessment were evaluated. Safety measures including standard blood tests were assessed (described as follows). 

Participants were randomized (1:1:1) and assigned to one of three groups. Block randomization was stratified by age, Mini-Nutritional Assessment (MNA^®^) and sex at enrolment and was carried out using library blockrand in R by Nestlé clinical development unit. The treatment group to which a subject was allocated was concealed to the participants, the support staff, the investigators and Nestlé, except for the quality specialists at the clinical development unit.

### 2.3. Supplementation

Participants were instructed to ingest dietary supplements consisting of one out of three experimental beverages together with 7 capsules (isocaloric—95 kcal) for 12 weeks on top of their twice weekly EMS treatment, as follows: (1) beverage with 20 g of carbohydrate (maltodextrin glucose syrup 21DE) + placebo capsules containing dextrin and medium chain triglycerides (CHO), (2) beverage with 20 g of whey protein isolate (95% whey, Prolacta^®^95) + placebo capsules (WPI) or (3) beverage with 20 g of WPI + hard capsules containing rutin (500 mg/day) + fish oil-derived ω3 fatty acids/curcumin in soft capsules (1.5 g/day fish oil type NAD from Sofinol that provided 18% eicosapentaenoic acid (EPA) and 7% docosahexaenoic acid DHA and 500 mg/day curcumin with 95% curcuminoids) (WPI + BIO). Curcumin and rutin were supplied by Sankyo Ltd. (Tokyo, Japan). The dose of protein used in this study is based on results from a previous study showing that 0.4 g of protein/kg body weight/serving is required to maximally stimulate muscle protein synthesis [36].

Depending on the group assignment, participants ingested the above-mentioned experimental beverages once per day, dissolved in 220 mL of water. On the days of EMS treatment, they were instructed to ingest the beverage just after EMS (maximal stimulation of protein synthesis). On the other days, participants drank the dietary supplement at the same time they were used to drinking it when receiving EMS. Participants were instructed to consume 7 capsules 3 times per day, 2 during breakfast, 3 during lunch and 2 during dinner for the duration of the study. Compliance was assessed by counting the left-over capsules at the end of the study. To help ensure the reliability of the capsule count, participants were asked to record the number of capsules ingested and asked to return any remaining pills at the end of the study.

### 2.4. Electrical Muscle Stimulation Procedures

The electrical muscle stimulation procedures have been fully described elsewhere [24,37]. Briefly, all participants received supervised EMS treatment 2 times a week during 12 weeks for 20 min with a specifically designed muscle stimulator (Auto Tens pro, Homer Ion Co. Ltd., Tokyo, Japan). Each time before the EMS, the physical condition of the participants was checked (resting heart rate and blood pressure). To be able to discriminate the specific effect of the beverage (and not the protein provided from the lunch), EMS was applied as early as possible in the morning (so that the beverage will be taken at least 1 h before the lunch) or later in the afternoon (at least 2 h after the end of the lunch). The participants were asked to attach belt-type electrodes around the waist and both knees and ankles to stimulate inner muscles as well as the gluteus maximus muscle, the quadriceps femoris, the hamstrings, the triceps surae and tibialis anterior muscles. EMS training was performed in a sitting position at rest to minimize the risk of developing lightheadedness, dizziness, falling and fainting caused by rapid movement. The stimulation intensity of EMS was regulated to the maximal tolerable level of each individual without discomfort. The stimulator current waveform was designed to produce co-contractions in the lower extremity muscle groups at a frequency of 20 Hz (muscle hypertrophy mode) with a pulse width of 250 μs. The duty cycle was a 5 s stimulation with a 2 s pause for a period of 20 min. Moreover, an exponential climbing pulse to reduce discomfort during muscle stimulation was used. For all participants, the total stimulation time and intensity was registered. There was no EMS on the day of testing in order to avoid muscle swelling and interference with the different tests.

Study endpoints were assessed before starting the treatment (W0) and again after 12 weeks (W12) for all variables except muscle morphology. All participants performed a Mini-Nutritional Assessment (MNA^®^) to evaluate their nutritional state before starting the treatment and after 12 weeks. This allows for classifying participants as normal, at risk of malnutrition or malnourished. All measurements were performed according to a standardized manner and participants were instructed not to take any drinks with caffeine the night before the testing. Participants were asked to come fasted for the blood samples. All measurements were done in the morning two days after the final training sessions.

### 2.5. Muscle Thickness

Muscle thickness of the rectus femoris (thigh) and gastrocnemius muscles (calf) was assessed by ultrasonography at baseline, 4 weeks, 8 weeks and 12 weeks of the experimental intervention. Strong correlations have been reported between muscle thickness measured by B-mode ultrasound and site-matched skeletal muscle mass measured by MRI [38]. Therefore, it is plausible to use the muscle thickness measurements to estimate muscle size and degree of muscle hypertrophy. Previous studies have shown the reliability of the ultrasound technique for measuring muscle thickness [39]. Also, the reliability of the ultrasonographic measurement was measured in this study. The intraclass correlation coefficients in rectus femoris (RF), vastus lateralis (VL) and calf (CA) muscles were 0.97, 0.96 and 0.99, respectively. The measurement of muscle thickness by ultrasonography was standardized as follows: all scans were carried out at baseline and every four weeks after treatment. Each subject was examined by the same operator, using a real-time scanner (SSD-900, ALOKA, Tokyo, Japan) with a 5 MHz broadband transducer. A water-based gel was applied to the probe before the imaging procedure. During imaging, the transducer was held perpendicular to the surface of the skin and special care was taken to avoid excessive pressure. The measurement site was at the thickest part of the muscles with standardized procedures using skeletal markers carefully located. It was measured at the half distance between the anterior superior iliac spine and the patella for RF and VL and at the half distance between the head of fibula and the lateral malleolus for CA. The probe was moved laterally to the midpoint between the greater trochanter and the tibia. The imaging and measurements was performed unilaterally with the participants in a sitting position for the quadriceps femoris and gastrocnemius muscles respectively, as these muscles were the main determinant for gait speed. The obtained images were stored on site and the entire dataset was analyzed later using the National Institute of Health (NIH) Image program. Data captured from each image was analyzed in a blinded fashion with respect to the subject and date information in order to avoid any experimental bias.

### 2.6. Physical Performance

Physical performance was assessed at baseline and 12 weeks of the experimental intervention. Methods for muscle strength measurements were reported elsewhere [40,41]. Briefly, isometric knee extensions were performed on a customized dynamometer mounting a force transducer (LU-100KSE; Kyowa Electronic Instruments, Tokyo, Japan). During contraction, both hip and knee joint angles were flexed at 90° (180° is fully extended). The maximal voluntary contraction (MVC) involved a gradual increase in knee extension force exerted by the knee extensor muscles from baseline to maximum in 2–3 s and then sustained at maximum for 2 s. The timing of the task was based on a verbal count given at a 1 s interval, with vigorous encouragement from the investigators when the force began to plateau. The participants performed at least two MVC trials of each knee with 2 min rest between trials. The highest MVC force was used for comparison. The average of left and right knees were used since there was no significant difference between left and right legs.

As part of the physical performance evaluation, gait speed was determined with the 6 m walking time test (seconds). A 6 m straight path was marked with tape on the floor, and using a custom-made trigger-linked stop-watch, the participants were asked to walk normally as in daily life during assessments. Usual walking aids (e.g., stick, walker) were allowed. Gait speed was evaluated three times for each subject and averaged.

Three minutes rest was provided between trials during the physical performance tests.

### 2.7. Body Composition and Plasma Measurements

Assessments of body composition (fat mass, lean mass, expressed as Kg and %) was conducted by bioelectrical impedance analysis (Tanita BC-118D, Tanita Ltd., Tokyo, Japan). Bioelectrical impedance analysis (BIA) is an alternative device for evaluating body composition, and has been used in clinical settings, as well as community and population-based studies. Previous research examined BIA in various experimental conditions and demonstrated its validity, reliability and reproducibility among different ethnic groups [42]. It was carried out with handheld leads together with flat foot sole electrodes with an excitation current of 500 microamperes at 50 kHz. The impedance value measured was used to calculate the lean mass and fat mass of the whole body, respectively. Participants were asked to urinate before the BIA measurement. All subjects came to the testing place after overnight fasting, thus ensuring the similar physiological conditions during the measurements while avoiding drinks, at least 2 h before the measurement.

The full battery of biochemical, hematological and coagulation parameters were analyzed at the clinical laboratory from SRL group (Kyoto, Japan, ISO15189) including: red and white blood cell count, markers of inflammation (CRP, transthyretin, fibrinogen and orosomucoid), blood chemistry of CPK, HDL and LDL cholesterol, albumin, total protein, triglycerides and blood test of coagulation parameters (platelet count, prothrombin time and partial prothrombin time) for safety control of the treatment.

### 2.8. Data Collection and Management

All data captured by the investigators were recorded on paper case report forms (CRF). Only blood biomarkers, ultrasonography and body composition results were generated as electronic files and were transmitted to the clinical data manager for loading and consolidation in the final database. All original CRF were sent to Nestlé and copies of all forms will remain at the study site. The CRF data was entered into a computer database (Clintrial 4.6^TM^) at Nestlé, Lausanne.

### 2.9. Statistics

Statistical analyses were carried out with SAS version 9.3. The muscle thickness was analyzed using a mixed model for repeated measures, with baseline measurement, sex, age at randomization and time as covariates. For muscle strength, the overall treatment difference was estimated with the help of the LSMEANS statement using a linear mixed model correcting for baseline, age and with a sex–treatment interaction term. An ANCOVA correcting for baseline value, sex, age at randomization and treatment group was used to evaluate the effect on walking speed and body composition. The time effect for muscle strength and gait speed was estimated by Wilcoxon tests. The effect of intervention on nutritional status (MNA^®^) was calculated using an ordered polytomous model. Odds ratios and confidence intervals for being at risk or improvement of nutritional status from baseline to 12 weeks were estimated by a proportional odds model, taking advantage of ordered categories. The incidence of adverse events (the percentage of participants having experienced at least one adverse events) during the treatment period was compared between the three randomized groups by exact Chi-Square test. Treatment differences for blood measurements was estimated by robust regression, correcting for baseline on log-transformed data. A *p* value ≤ 0.05 was considered as being statistically significant. Data are expressed as means ± SEM.

Since it was a first proof of concept study, no formal sample size calculations were performed. The number of participants to recruits was set to 15 participants per treatment group.

## 3. Results

### 3.1. Subject Characteristics and Compliance

**Figure 1:**
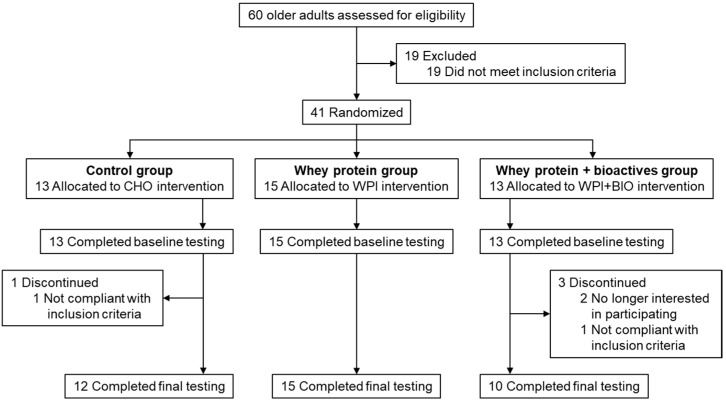
Flow of study participants: elderly subjects with limited mobility treated with a combination of electrical muscle stimulation (EMS) and nutritional supplement: CHO, WPI or WPI + BIO for 12 weeks. BIO: bioactives, CHO: carbohydrates, WPI: whey protein isolate.

A total of 37 subjects (12 subjects (92%) in the CHO group, 15 subjects (100%) in the WPI group and 10 subjects (77%) in the WPI + BIO group) completed the study and were included in the statistical analysis. Per-protocol populations for analysis included participants who completed the final testing. The flow of study subjects is shown in Figure 1 and baseline characteristics of the subjects who completed the study are described in Table 1. The average compliance of subjects who completed the study, as judged by the leftover pill count, was 97.2% in the CHO group, 96.8% in the WPI group and 97.4% in the WPI + BIO group.

### 3.2. Muscle Thickness

**Figure 2:**
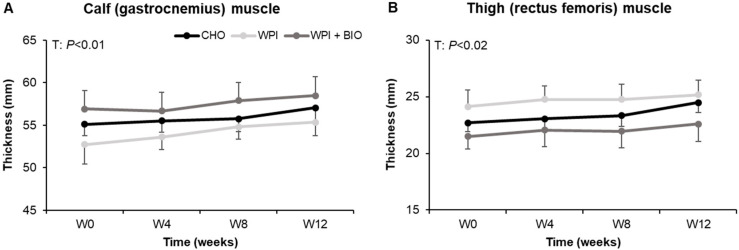
Thickness of (**A**) calf and (**B**) thigh muscles assessed by ultrasonography in elderly subjects with limited mobility treated with a combination of EMS and nutritional supplement: CHO, WPI or WPI + BIO for 12 weeks. Values are means ± SEM (*n* = 10–15). The time statistical effects (T) are reported for each muscle (Wilcoxon tests). BIO: bioactives, CHO: carbohydrates, WPI: whey protein isolate.

Calf (Figure 2A) and thigh (Figure 2B) muscles’ thickness were significantly (time effect, *p* = 0.008 and *p* = 0.018, respectively) increased by 3% to 5% in all treatment groups. The size of the muscle was significantly higher in all groups after 12 weeks of intervention but no statistical difference was observed among CHO, WPI and WPI + BIO groups (no treatment by time interaction and treatment effect). The same effect was observed for the cross-sectional area (data not shown).

### 3.3. Physical Function

**Figure 3:**
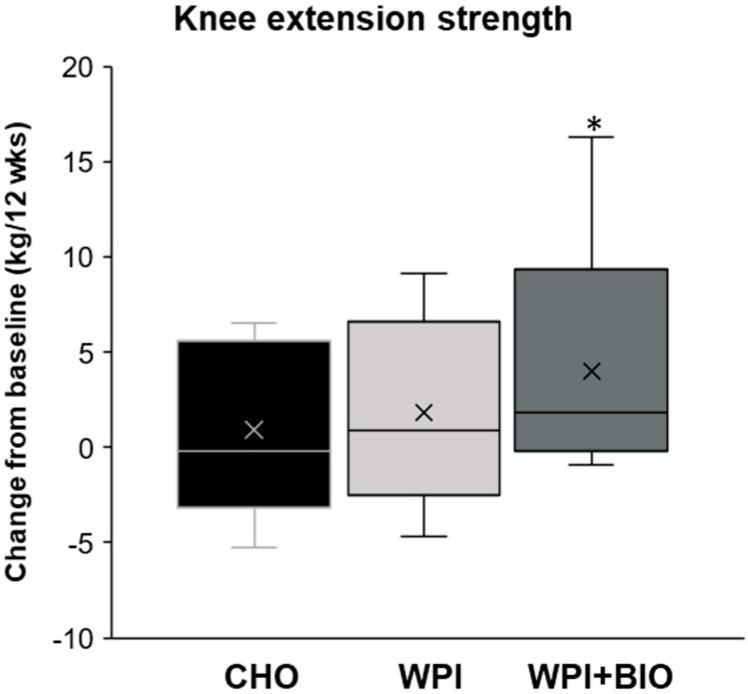
Change from baseline for muscle knee extension strength in elderly subjects with limited mobility treated with a combination of EMS and nutritional supplement: CHO, WPI or WPI + BIO for 12 weeks. In the box plots, the boundary of the box closest to zero indicates the 25th percentile, a line within the box marks the median, a cross within the box marks the mean, and the boundary of the box farthest from zero indicates the 75th percentile. Whiskers above and below the box indicate the 10th and 90th percentiles. (*n* = 10–15). * significantly different from CHO, *p* < 0.05 (linear mixed model). BIO: bioactives, CHO: carbohydrates, WPI: whey protein isolate.

Muscle knee extension strength was significantly influenced by WPI + BIO supplementation (Figure 3 and Table 2). The +2.8 kg change from baseline (Figure 3) was significantly higher in WPI + BIO supplementation compared to the CHO group (*p* = 0.025), leading to a 13% improvement in muscle strength in the WPI + BIO group along the 12 weeks of treatment (5% and 6% improvement in CHO and WPI groups, respectively). WPI supplementation was not significantly different than CHO and WPI + BIO. In line with these results, there was a significant time effect only in the WPI + BIO group (Table 2; *p* = 0.042).

Gait speed estimated using a 6 m walking time test (Table 3) increased significantly by 8% from baseline to 12 weeks of supplementation (time effect) in the WPI + BIO group (*p* = 0.032). By contrast, it was not significantly increased in either the CHO or WPI groups (around 3% increase from baseline). However, there was no significant treatment by time interaction or treatment difference between groups.

### 3.4. Body Weight and Composition

The body weight of the participants remained stable following the three separate nutritional interventions compared to baseline (47.7 ± 3.3, 49.7 ± 2.8 and 53.5 ± 3.0 kg after 12 weeks of CHO, WPI and WPI + BIO supplementations, respectively).

WPI or WPI + BIO supplementations did not have any effect on total lean (Table 4) and fat mass (data not shown) compared to CHO supplementation.

### 3.5. Nutritional Assessment

The change in prevalence of participants classified as normal, at risk of malnutrition or malnourished by MNA^®^ was not significantly affected by nutritional supplementations (Table 5). 50% of the participants improved their status over the 12 weeks of intervention in the WPI + BIO group (5 out of 10 participants) compared to 25% (3 out of 12) and 13% (2 out of 15) in the CHO and WPI groups, respectively. However, the odds ratio for improvement or being at risk after intervention was not affected by the nutritional supplementation.

At baseline, blood albumin level was 4229 ± 321, 4368 ± 341 and 4308 ± 333 mg·dL^−1^ in CHO, WPI and WPI + BIO respectively, while at week 12 it was 4529 ± 415, 4524 ± 471 and 4342 ± 406 mg·dL^−1^.

Circulating total protein level was 7.35 ± 0.44, 7.50 ± 0.53 and 7.37 ± 0.34 g·dL^−1^ in CHO, WPI and WPI + BIO respectively, at baseline. At week 12, it was 7.54 ± 0.49, 7.71 ± 0.44 and 7.52 ± 0.39.

There was no difference between groups.

### 3.6. Adverse Events

There were no whey protein, fish oil and polyphenols ingestion-related changes in red and white blood cell count, markers of inflammation (CRP, transthyretin, fibrinogen and orosomucoid), blood chemistry of CPK, HDL and LDL cholesterol, albumin, total protein and triglycerides at baseline and at week 12 (data not shown). Similarly, blood test of coagulation parameters (platelet count, prothrombin time and partial prothrombin time) measured at baseline, 2, 6 and 12 weeks did not show significant group differences. There was no significant difference among groups for serious adverse events. The most commonly reported side effect for all participants who initiated the study was gastrointestinal symptoms, which was reported by 15.4% of the participants (*n* = 2) in the CHO group, 26.7% (*n* = 4) in the WPI group and 23.1% (*n* = 3) in the WPI + BIO group.

## 4. Discussion

This double-blind, randomized trial in elderly adults with mobility limitations is the first one testing the impact of a specific blend of polyphenols and omega-3 (ω3) fatty acids on top of whey protein combined with EMS treatment. Muscle thickness significantly increased in all groups but there were no statistical differences related to nutritional supplementation. By contrast, the change in muscle knee extension strength was significantly improved versus placebo in the WPI + BIO group with curcumin, rutin and ω3 on top of EMS. On top of this 13% increase in muscle strength, the largest improvement in gait speed (8%) was observed in the WPI + BIO group compared to the other groups. Despite the positive effect on muscle strength and gait speed, there were no significant changes in body composition and nutritional status.

The muscle thickness of the leg of the participants was significantly improved by 3% to 5% in all treatment groups, suggesting that this effect could be due to EMS treatment only. The beneficial impact of resistance or endurance training on muscle mass and function is highlighted in many studies, suggesting that the potential anabolic response to exercise still remains despite the reduced metabolic response to nutrients generally observed in this population [21,22]. However, exercise programs may be difficult to carry out effectively in frail or bedridden individuals. These results provide supportive evidence that EMS could be a valuable alternative and efficient solution to counteract age-related loss of muscle health in elderly adults. This is in line with a study showing increase of lean body mass in women at risk of sarcopenia who were treated with whole-body electromyostimulation [27]. There are limited studies assessing the effect of EMS alone on muscle thickness in elderly subjects. The increase in muscle thickness was around 7% after 8 weeks of EMS treatment in elderly subjects [43] and 5% in intensive care unit patients [44]. These results are in line with the effect of EMS we observed. It has also been shown that EMS can also improve physical performance, e.g., strength, time up and go and short physical performance battery in healthy elderly adults [28,29,31]. Depending on the exercise program, it was shown that EMS had identical or even greater benefits compared to voluntary muscle training during a phase of reduced activity [25,45] or after an immobilization period [26]. One mechanism of action that could explain this observed increase in muscle size is the stimulation of muscle protein synthesis [46] and the decrease of protein degradation [31,46] observed with EMS treatment. 

In our study, the WPI group did not demonstrate any benefit as compared to the CHO group. The short-term effects of whey protein were well studied and shown to stimulate postprandial muscle protein accretion in elderly adults [47]. This is related to the activation of the muscle protein synthesis signaling pathway in elderly adults [47,48]. The translation of this short-term benefit on muscle mass and functionality is controversial. Tieland et al. [12] demonstrated a beneficial effect of protein on physical performance in frail elderly adults but others did not demonstrate any long-term benefit [49]. Indeed, two studies have shown a benefit on muscle mass and function in sarcopenic elderly adults after long-term intervention with whey protein supplementation, although it may be due to the added leucine and vitamin D [50] or on top of exercise [51]. The observed effect related to the combination of whey protein and exercise has not been confirmed in well-nourished healthy older woman [52,53] nor in mobility-limited older adults [54]. Our study also failed to show a benefit with the whey protein supplementation alone. We can therefore speculate that protein alone is not sufficient to have a long-term beneficial effect on muscle mass and functionality.

The main finding of the present study was a significant 13% change from baseline in muscle knee extension strength observed in the whey protein group supplemented with ω3, curcumin and rutin compared to the change in the carbohydrate group. WPI supplementation had the same effect as the CHO. On top of this effect of WPI + BIO, there was an 8% (0.09 m·s^−1^) improvement in gait speed, which is clinically meaningful. Previous studies show that clinically meaningful gait speed improvements (0.05 to 0.14 m·s^−1^) have been associated to better physical performance and survival rates [55,56]. Since all groups were treated with electrical muscle stimulation, our results demonstrate that the nutritional supplementation with WPI + BIO had a specific benefit during the 12 weeks, which could potentially be beneficial independently of EMS. However, it is possible that EMS treatment induced a permissive anabolic environment improving the sensitivity of the muscle to the specific nutrient effect. Indeed, it was previously shown that exercise is capable of boosting the beneficial effects of curcumin and fish oil [57].

Omega-3 fatty acids are emerging ingredients for muscle benefits. It significantly improved muscle functionality (walking speed and chair rising) in elderly woman after 90 days [20] and 6 months [18] but there was no effect on the preservation of muscle mass. One study has shown a significant 4% increase in lean mass and a decrease in time up and go speed after 12 weeks of fish oil supplementation in healthy community-dwelling women [19]. Less is known about the effect of polyphenols on muscle function and health. One open-label clinical study assessing the effect of curcumin supplementation on muscle functionality in elderly adults reported improved muscle strength and physical performance when combined with an exercise program but this study was not a randomized blind trial [16]. 

Different hypotheses can be postulated to explain the mechanism of action for the present results observed on muscle strength after WPI + BIO ingestion. First, muscle strength can be influenced not only by the quantity of the muscle (muscle cross-sectional area), but also by the quality of the muscle and fiber types [58]. It was shown that curcumin improves the recovery of cross-sectional fiber area after unilateral hindlimb immobilization in preclinical studies [59]. Increased knee extension strength might also be due to improved muscle metabolism. Higher muscle protein metabolism sensitivity to anabolic factors (amino acids and insulin) and stimulation of the mTOR (mammalian target of rapamycin) signaling pathway were observed after ω3 supplementation in older adults [17]. Preclinical studies with curcumin, rutin and other antioxidants have demonstrated improvement in muscle protein synthesis [14,15] or decreased muscle protein breakdown after immobilization [59] and in old rats [14]. Finally, curcumin, rutin and ω3 could also help to manage different factors like inflammation, oxidative stress and mitochondrial dysfunction. Indeed, the combination of curcumin and exercise increased muscle mitochondrial biogenesis in rats [60] and four months of ω3 supplementation reduced skeletal muscle oxidative stress by reducing mitochondrial reactive oxygen species production in 75-year-old older adults [61]. Mitochondrial function has also been improved after 10 weeks of eicosapentaenoic acid (ω3 fatty acid) supplementation in old mice [62].

Our study had some limitations: as a first proof of concept study, there was a small number of participants and the results need to be confirmed in a larger clinical study. In addition, we were not able to recruit equal amounts of males and females in each group. Moreover, we did not control or monitor dietary intake of our participants, although they were asked to maintain their habitual dietary intakes throughout the entire study period. This is a major limitation but there was no difference between groups for blood albumin and total protein level. Moreover, the MNA is assessing the nutritional status of the participants with questions related to the frequency of consumption of dairy products, eggs, meat, fish, vegetables, etc., and there was no difference between groups. MNA was used for randomization, thus limiting the risk of having important differences related to nutritional status. Lastly, we did not have a specific arm testing the WPI + BIO without EMS.

## 5. Conclusions

In conclusion, we showed that the muscle size was increased in all groups and this could be explained by EMS treatment. WPI + BIO administration in combination with EMS elicits a significant increase of muscle knee extension strength and the largest improvement in gait speed (8%) after 12 weeks of treatment in elderly adults with limited mobility. This effect was not observed in the group supplemented with whey protein only. The functional improvement observed might therefore be related to physiological benefits related to a positive interaction between polyphenols, fish oil and protein. The underlying mechanism of action needs to be elucidated but such combinations of ingredients may help to manage the oxidative stress that could interfere with mitochondrial function or help to reverse the anabolic resistance. Thus, a multimodal approach combining such nutritional combination and exercise induced by EMS may provide an innovative treatment for limiting the development of sarcopenia with aging.

## Figures and Tables

**Table 1:** Baseline characteristics of the elderly subjects with limited mobility treated with a combination of EMS and nutritional supplement: CHO, WPI or WPI + BIO for 12 weeks.

	CHO (*n* = 12)	WPI (*n* = 15)	WPI + BIO (*n* = 10)
Age (years)	78 ± 2	78 ± 1	76 ± 2
Sex, *n* (%)			
Male	2 (16)	3 (20)	2 (20)
Female	10 (84)	12 (80)	8 (80)
Body mass and composition			
Body mass (kg)	48.3 ± 2.9	49.7 ± 2.7	52.5 ± 2.9
Body mass index (kg/m^2^)	20.8 ± 0.8	21.3 ± 0.9	22.5 ± 1.1
Body fat (%)	29.3 ± 2.0	28.7 ± 2.6	29.7 ± 2.8
Thigh muscle thickness (mm)	22.7 ± 0.8	24.1 ± 1.5	21.5 ± 1.1
Calf muscle thickness (mm)	55.1 ± 1.4	52.7 ± 2.3	56.9 ± 2.1
Physical function			
Knee extension strength (kg)	19.8 ± 1.3	25.2 ± 2.2	22.4 ± 3.0
Gait speed (m/s)	1.16 ± 0.08	1.11 ± 0.09	1.10 ± 0.14

Results are expressed as means ± SEM. BIO: bioactives, CHO: carbohydrates, WPI: whey protein isolate.

**Table 2:** Muscle knee extension strength of elderly subjects with limited mobility treated with a combination of EMS and nutritional supplement: CHO, WPI or WPI + BIO for 12 weeks.

	CHO (*n* = 12)	WPI (*n* = 15)	WPI + BIO (*n* = 10)
Week 0 (kg)	19.8 ± 1.3	25.2 ± 2.3	22.4 ± 3.0
Week 12 (kg)	20.8 ± 1.4	26.8 ± 2.2	25.3 ± 3.5 *
*Time effect* ^1^	*p* = 0.244	*p* = 0.064	*p* = 0.042

Results are expressed as means ± SEM. ^1^ Wilcoxon tests. * Significantly different from week 0. BIO: bioactives, CHO: carbohydrates, WPI: whey protein isolate.

**Table 3:** Gait speed determined with the 6 m walking time test of elderly subjects with limited mobility treated with a combination of EMS and nutritional supplement: CHO, WPI or WPI + BIO for 12 weeks.

	CHO (*n* = 12)	WPI (*n* = 15)	WPI + BIO (*n* = 10)	*Treatment Effect* ^1^
Week 0 (m/s)	1.16 ± 0.08	1.11 ± 0.09	1.10 ± 0.14	NS
Week 12 (m/s)	1.19 ± 0.06	1.16 ± 0.08	1.19 ± 0.13 *	NS
*Time effect*	*p* = 0.735	*p* = 0.454	*p* = 0.032 ^2^	

Results are expressed as means ± SEM. ^1^ ANCOVA. ^2^ Wilcoxon tests. * Significantly different from week 0. BIO: bioactives, CHO: carbohydrates, WPI: whey protein isolate. NS, not significant (*p* > 0.05).

**Table 4:** Total lean mass determined by bioelectrical impedance analysis of elderly subjects with limited mobility treated with a combination of EMS and nutritional supplement: CHO, WPI or WPI + BIO for 12 weeks.

	CHO (*n* = 12)	WPI (*n* = 15)	WPI + BIO (*n* = 10)	*Treatment Effect* ^1^
Total lean mass (%)				
Week 0	70.8 ± 2.3	71.2 ± 2.6	70.9 ± 2.6	NS
Week 12	70.8 ± 2.5	71.3 ± 2.5	69.2 ± 2.6	NS
Total lean mass (kg)				
Week 0	33.7 ± 5.8	35.1 ± 7.8	37.1 ± 7.3	NS
Week 12	34.2 ± 5.7	35.0 ± 7.4	36.1 ± 6.5	NS
*Time effect*	*p* = 0.244	*p* = 0.754	*p* = 0.906	

Results are expressed as means ± SEM. ^1^ ANCOVA. No differences were detected between groups. BIO: bioactives, CHO: carbohydrates, WPI: whey protein isolate. NS, not significant (*p* > 0.05).

**Table 5:** Number of elderly subjects with limited mobility classified at normal nutritional state, at risk of malnutrition or malnourished with the Mini Nutritional Assessment (MNA^®^) before and after treatment with a combination of EMS and nutritional supplement: CHO, WPI or WPI + BIO for 12 weeks.

	CHO (*n* = 12)	WPI (*n* = 15)	WPI + BIO (*n* = 10)
Week 0			
Normal nutritional state	6	8	4
At risk of malnutrition	6	6	5
Malnourished	0	1	1
Week 12			
Normal nutritional state	9	9	7
At risk of malnutrition	3	6	3
Malnourished	0	0	0

Results are expressed as number of subjects. BIO: bioactives, CHO: carbohydrates, WPI: whey protein isolate.
